# Nutritional Strategies for Olympic Biathletes: A Practical Review

**DOI:** 10.3390/nu17213385

**Published:** 2025-10-28

**Authors:** Mateusz Gawelczyk, Magdalena Kaszuba, Miroslav Petr

**Affiliations:** 1 Institute of Sport Sciences, The Jerzy Kukuczka Academy of Physical Education in Katowice, 40-065 Katowice, Poland; m.kaszuba@awf.katowice.pl; 2 Faculty of Physical Education and Sport, Charles University, 162 52 Prague, Czech Republic; miroslav.petr@ftvs.cuni.cz

**Keywords:** biathlon, supplements, diet, sports nutrition, athlete performance

## Abstract

Biathlon is a winter Olympic sport that combines high-intensity cross-country skiing with precise rifle shooting. These dual demands require athletes to develop exceptional aerobic capacity while maintaining fine motor accuracy under physiological stress. Despite its complexity, nutritional strategies in biathlon remain under-explored and recommendations are often extrapolated from related endurance sports. This narrative review aims to summarise existing knowledge on nutrition in biathlon, highlight sport-specific challenges and identify areas for future research. The main findings indicate that biathletes face very high energy demands, with daily expenditure exceeding 7000 kcal during intensive training. Carbohydrates are the primary fuel source, with intake recommendations based on training intensity and duration (6–12 g/kg/d). Furthermore, protein is essential for muscle repair, recovery and adaptation. To achieve the recommended intake of 1.6 g/kg/day, it is advisable to consume meals containing approximately 0.3 g/kg of high-quality protein every three to four hours. Given the frequency of training sessions, effective recovery strategies are important in biathlon. When recovery is a priority, biathletes should consume a meal comprising protein (approximately 0.3 g/kg) and carbohydrates (approximately 1.2 g/kg) before key training sessions. Micronutrient and vitamin deficiencies are not commonly observed in biathletes due to their high calorie intake. However, concerns regarding iron and vitamin D are common among endurance athletes due to the high risk of low energy intake, diets lacking in iron, and insufficient exposure to sunlight. On the day of the race, it is recommended that biathletes plan their meals to ensure that they meet their nutritional needs and begin recovery as soon as possible after the race is over. Biathletes may use specific supplements to enhance performance and health during preparation and competition. However, it is important to note that some supplements that improve performance may harm shooting accuracy. Current guidance is provisional, and future research should adopt a dual-performance framework that evaluates both endurance output and shooting precision under realistic competition conditions.

## 1. Introduction

The biathlon has been part of the Winter Olympic Games since 1960. An intermittent endurance sport combines cross-country ski skating at a designated distance separated by rifle shooting. It demands high aerobic capacity for skiing and proficiency in shooting. Athletes require extensive training to enhance their physical abilities, perfect their ski skating techniques, and maintain shooting accuracy within brief timeframes. We distinguish between individual sprints, long-distance races, mass start and pursuit races for men and women and relay races for men, women, and mix. Individual elite senior events last between 20 and 48 min, comprising two to four shooting stages. Relays last between 16 and 20.5 min, while the single mixed relay lasts around 6 to 7 min [1,2]. Race programmes consist of 3–7 race days for World Cup events and 9 race days for World Championships or Olympic Games. Overall performance in the biathlon is complex and determined by multiple factors, including skiing speed, time spent at the shooting range, shooting duration, and shooting accuracy. The demands of the Olympic biathlon competition have been reviewed elsewhere [1].

While information on race formats and training calendars helps us to understand the sport, a more important question is why a review focusing on nutrition is necessary. Compared with other endurance sports, biathlon presents unique nutritional challenges. Firstly, energy demands are extremely high (daily expenditure can exceed 7000 kcal during training camps), yet athletes must also maintain lean body mass and low body fat in order to optimise their skiing efficiency. Secondly, biathlon requires rapid recovery across consecutive race days, often with less than 24 h between competitions. This makes nutrition a key factor in sustained performance. Thirdly, unlike in pure endurance sports, performance depends not only on maximal aerobic capacity, but also on the ability to maintain fine motor accuracy when shooting. This dual demand raises unique nutritional questions: a strategy that enhances aerobic capacity may impair fine-motor control. For example, caffeine is well established to improve endurance performance, but recent evidence indicates that it can reduce shooting accuracy in biathlon. Such interactions highlight the urgent need for biathlon-specific studies, as extrapolating from other sports may not always provide safe or optimal guidance. Fourthly, athletes often compete or train in cold, high-altitude environments, which increases energy expenditure and alters substrate use, thereby exacerbating the risk of dehydration and iron deficiency.

Despite these unique demands, no prior review has synthesised evidence-based nutritional strategies specifically for biathlon. Current recommendations are often extrapolated from studies on cross-country skiing or other endurance sports, but these may not fully capture the complexity of biathlon. Therefore, this review aims to critically evaluate current knowledge on energy and macronutrient requirements, micronutrient considerations, hydration, supplementation and recovery strategies in the context of biathlons. Furthermore, the objective is to identify knowledge gaps and provide practical recommendations for athletes, coaches, and other practitioners in this field.

To our knowledge, this is the first review to comprehensively address the nutritional needs of biathletes in relation to their specific physiological, technical, and environmental requirements. It provides evidence-based recommendations and highlights important areas for future research.

### Study Design

This article is a narrative review. We selected this typology because of the very limited number of biathlon-specific nutrition and supplementation studies, which precludes a systematic review or meta-analysis. A narrative approach allows integration of evidence from related endurance sports (e.g., cross-country skiing, cycling, triathlon) where appropriate, combined with practical insights into the dual performance demands of biathlon.

This review was conducted as a narrative synthesis. Using the search terms ‘biathlon AND nutrition’, ‘biathlon AND supplementation’, and ‘biathlon AND physiology’, we searched PubMed and Google Scholar for studies published by December 2024. Due to the limited number of biathlon-specific publications, studies from closely related endurance sports (e.g., cross-country skiing, cycling and triathlon) that addressed topics relevant to biathlon training or competition were also included. The inclusion criteria comprised peer-reviewed articles in English reporting on nutrition, supplementation, energy expenditure, recovery or environmental challenges in endurance sports. Non-peer-reviewed materials, case reports without nutritional relevance and studies unrelated to endurance or precision performance were excluded.

The review sub-topics (energy requirements, macronutrients, micronutrients, physique, race-day nutrition, cold and altitude, and supplements) were chosen in advance based on the main areas highlighted in international position statements on sports nutrition. These were then adapted to reflect the unique dual requirements of biathlon: high aerobic output during skiing combined with neuromuscular precision and psychological control during shooting.

## 2. An Overview of the Periodized Training and Competition Calendar

The preparation season for biathletes typically begins in May and continues until the beginning of November. During this period, athletes typically complete 70–80% of their annual training programme [1]. Competitors engage in a substantial volume of physical training to achieve excellence in biathlon. The training regimen typically incorporates endurance training at a range of intensities, from low to high, with the latter reaching approximately 5–6% of maximum heart rate. Strength and speed training, comprising approximately 10% of the total training volume, are also included. The average training duration for successful biathletes ranges between 700 and 900 h per year [1,3,4]. Senior biathletes engage in 8–18 h of training per week, with variations depending on the season [3,5]. Most training sessions (75–80%) are characterised by low-intensity training, with moderate- and high-intensity training accounting for approximately 10% of the total training time.

The biathlon competition season typically starts in November and continues until the season’s closing in March. The biathlon season includes nine World Cup events and the World Championships. Each World Cup consists of two or three individual races for both men and women and one or two relays. During the World Championships, all four individual races are held for both men and women, as well as three relays [6].

## 3. Nutrition for Training

The following sections primarily refer to elite and highly trained biathletes, as most available data and applied practices derive from this population. While general principles may also inform junior athletes, their absolute requirements may differ due to lower training loads and competition demands.

### 3.1. Energy Requirements

Biathletes complete a very differentiated training programme, consisting of skiing, roller skiing or cycling, shooting sessions, and gym or cross-training, which demands wide energy needs. Moreover, the energy requirements depend on training volume and intensity, growth, goals (e.g., change in body mass/fat mass) and other lifestyle activities. In biathlon, training and competition are entirely different in duration and intensity. While competition for the biathletes lasts around 20–50 min with high-intensity effort separated by shooting, trainings usually have lower intensity but lasts around 2–4 h. To date, no single study accesses energy expenditure in biathletes. A study conducted on cross-country skiers show that the daily energy expenditure during the preparation period, which often includes two training sessions per day, is 4800–6000 kcal/d [7]. In contrast, during intense, on-snow training, it has been observed to be 950–1900 kcal/d higher [7]. The respective figures for male and female athletes are approximately 7149 kcal/day and 4357 kcal/day [8]. Cross-country skiers are recommended to consume 55–75 kcal/kg/d, with the quantity depending on the individual’s needs [9]. It is important to note that, although cross-country skiers and biathletes both run on skis, the additional weight of the rifle on the back of the biathlete’s back introduces a different set of physical demands. Compared to cross-country skiing, carrying a rifle (which represents an additional load of approximately 4 kg) while skiing incurs an average cost of approximately 7% higher [10]. Although extrapolation from cross-country skiing provides a useful starting point, the lack of direct measurements of energy expenditure in biathlon, especially under race conditions, remains a critical gap.

As is the case in numerous other endurance sports with considerable energy demands, there is also a risk of low energy availability (LEA) in biathlon. This arises from a discrepancy between energy expenditure and energy intake, whereby the former exceeds the latter to such an extent that it cannot provide the requisite energy to support the functions necessary for maintaining optimal health and performance [11]. A consequence of prolonged energy deficiency in athletes (EDA) is relative energy deficiency in sports (RED-S). REDs have numerous adverse implications for health and performance. These include impaired immunity, reproductive function, glycogen resynthesis, and cardiovascular and haematological health issues. Additionally, REDs can cause sleep disturbances, impair growth and development, and reduce training response and performance, including endurance, power and strength, or motivation [12]. The only study conducted on cross-country skiers indicates that athletes encounter challenges in meeting their energy and carbohydrate requirements during a training camp. Despite the mean energy availability being 40.3 ± 17.3 kcal/kg FFM/d, which is above the previously established universal cut-off limit for women (>30 kcal/kg FFM/d), the range for athletes in this research was 11.1–78.4 kcal/kg FFM/d [13]. To determine the risk of LEA in athletes with greater expediency, medical practitioners or nutritionists may utilise validated questionnaires, namely the LEAF-Q and LEAM-Q, which have been developed for women and men, respectively [14,15].

### 3.2. Carbohydrate Requirements

Most biathletes’ key training sessions are performed at intensities highly dependent on carbohydrate (CHO) based fuels for muscle metabolism. Biathletes typically undertake two training sessions per day, including roller skiing, cycling, cross-country skiing, strength training, cross or yoga. The diverse nature of these sessions gives rise to differing carbohydrate requirements. It is, therefore, essential that nutrition aligns with the specifics of the training plan, considering each session’s duration, intensity and objective, to provide the necessary fuel for the work required. According to current recommendations, biathletes should consume 6–12 g/kg/d of carbohydrate, depending on their training programme [16]. This should facilitate the replenishment of muscle glycogen when required. Table 1 and Table 2 demonstrate the carbohydrate-dependent training programmes and how the total carbohydrate intake could be distributed over the sample week and within a day to guarantee high carbohydrate availability for the most important training sessions during the summer and winter training periods. These tables illustrate an adapted framework for nutritional periodisation in biathlon. While they are based on established endurance nutrition guidelines, they also consider sport-specific factors, such as the repeated transitions between high-intensity exertion and precise shooting. Some training sessions may be carried out with a reduced carbohydrate availability to expose the muscle to a greater training stimulus and, consequently, promote a more significant metabolic adaptation. For more details see [13,14]. It is important to note that training with low glycogen availability is associated with an increased risk of illness [17], injury [18], and overtraining [19].

Furthermore, reduced training intensity and/or volume [20,21] and impaired post-exercise muscle protein synthesis regulation [22] have been observed. Moreover, a recent meta-analysis of nine studies examining the long-term benefits of carbohydrate periodisation on performance outcomes indicates that this approach may not consistently enhance performance compared to training with high carbohydrate availability [23]. The collective evidence suggests that training with reduced carbohydrate availability may be constrained due to reduced training duration and/or intensity. However, this may not apply to individuals seeking the most time-efficient training. Therefore, although these strategies can be used selectively, they cannot be routinely recommended to biathletes until studies specific to the sport confirm their long-term benefits.

During the season, biathletes are required to spend a significant amount of time at training camps or travelling to and from competitions. This often results in limited access to the kitchen facilities, which can impact their ability to meet dietary recommendations. To ensure adequate carbohydrate intake, it is essential to incorporate carbohydrate-rich foods at meals when possible and provide high-carbohydrate snacks between meals, particularly on days with elevated energy expenditure and/or intense physical activity. Furthermore, it is important to guarantee the intake of carbohydrates both before and during exercise, as well as during the subsequent recovery period.

The duration of biathlon training sessions typically exceeds 90 min, which, per the recommendations for endurance athletes, necessitates the provision of carbohydrates [24]. Based on the recommendation for athletes, biathletes should consume 30–90 g of carbohydrates, depending on the training intensity and duration [25]. The primary carbohydrate source for athletes undertaking exercise should be an isotonic drink, which also improves hydration status. During winter, isotonic drinks might be kept warm in insulated containers. In addition, biathletes may consume an energy bar or gel, such as a banana or jelly bar, during short breaks in training. The current studies indicate no significant difference in oxidation rates between isotonic drinks, bars, and gels [26,27]. Therefore, athletes can select the product that aligns with their preferences. The ingestion of carbohydrates during exercise benefits exercise performance [28]. Moreover, consuming carbohydrates during prolonged and/or high-intensity exercise may help to counteract the reduction in immune system activity and reduce the risk of illness, which is particularly important for winter sports [29].

A typical biathlete engages in two training sessions daily, with an interval of 4–6 h between sessions. This gap is shorter than in other disciplines, such as triathlon (5–7 h) [30] and cycling, where there is usually only one training session a day. The complete replenishment of glycogen stores occurs within a 24 h [31]. Given the brief interval between training sessions, biathletes are advised to prioritise recovery immediately following each session. It is recommended that 1.2 g/kg of CHO be consumed immediately after exercise [32]. In instances where the CHO intake is insufficient, administering 0.3 g/kg of protein has been demonstrated to enhance glycogen resynthesis rates [33]. Moreover, whey hydrolysate appears to be more effective in this regard than other types of protein and the production procedure [34,35]. The initial timing of the post-exercise meal is of particular significance, as evidence suggests that the rate of muscle glycogen synthesis is accelerated during the initial 2 h following exercise compared to the subsequent hours [36]. From a practical standpoint, a recovery shake is a suitable option for athletes needing a post-training refuelling strategy. The shake should contain milk, banana, protein powder, and other low-fibre fruits or cereals tailored to the athlete’s needs. This can be easily transported and consumed immediately following training.

### 3.3. Protein Requirements

Protein is typically linked to high muscle mass and is regarded as the most crucial macronutrient for power sports. Nevertheless, endurance athletes must also prioritise protein intake for optimal training adaptations. Current research indicates that 1.6 g/kg of body mass per day is adequate for muscle development [37,38,39]. Moreover, it is advised that biathletes engaged in twice-daily training programmes adhere to the current guidelines for the timing of protein intake. This should be at a rate of 0.3 g/kg BM of protein (~20–25 g of high-quality protein) immediately following a key training, resistance exercise, or race. This ensures that the early protein synthetic response to the training stimulus is optimised [40,41]. It is noteworthy that the intake of protein (approximately 20–25 g) while carbohydrate intake is inadequate (<1.2 g/kg) has been observed to enhance glycogen resynthesis [33]. It should be noted that the recommended intake of ~1.6 g·kg^−1^·d^−1^ is extrapolated from endurance athletes in general. Furthermore, no study has investigated the timing or dosage of protein intake specifically in biathlon, nor has any study verified whether the timing or distribution of protein intake affects recovery and shooting performance in biathletes. Given the dual demands of muscle recovery and maintaining lean body mass throughout a long season, biathletes may benefit from a more strategic approach to protein intake. This hypothesis requires direct testing.

### 3.4. Micronutrient and Vitamins Requirements

Vitamins and minerals are essential nutrients for maintaining optimal performance. It is widely acknowledged that deficiencies in these nutrients can adversely affect overall health and bodily functions. However, deficiencies are not commonly observed due to the high caloric intake typical of biathletes. It appears that, provided athletes consume a typical diet, they obtain sufficient nutrients. The primary concerns regarding micronutrient status are generally limited to iron and vitamin D. These findings are typically associated with restrained eating practices and inadequate food variety.

Iron is a micronutrient that is essential for oxygen delivery within the body. A decline in iron levels has been observed to impair athletic performance, with the underlying mechanism thought to be related to the role of iron in oxygen transport [42]. Furthermore, iron deficiency is frequently observed in athletes engaged in endurance sports, with female athletes demonstrating a higher prevalence of this condition than males [43,44]. The reason for that is complex, but the most common include low energy intake, vegetarian diets and endurance exercise [44,45]. It is therefore crucial to monitor athletes’ iron status regularly through specific blood markers, including ferritin, haemoglobin concentration and transferrin saturation [46]. It is a common practice in endurance sports to provide additional iron supplementation even when the level is within the recommended range. However, no evidence suggests that such supplementation improves endurance performance in athletes who do not have iron deficiency [47,48,49]. Athletes must pay close attention to their iron intake, ensuring they consume sufficient quantities of meat, fish, legumes, dark green vegetables, nuts and seeds. The current recommended intake for elemental iron is 8 mg for males and 18 mg for females [50]. Nevertheless, recommendations for the general population may prove inadequate for athletes [46].

A suboptimal level of vitamin D is a prevalent phenomenon among the general population on a global scale. It is typical for levels to be lower during winter and among athletes who train indoors instead of outdoors. Given the role of vitamin D as a gene modulator, it is recommended that the level be sustained at >50 nmol/L [51] to maintain optimal immune function, bone health and the rehabilitation process. If exposure to sunlight is inadequate, as well as dietary intake, athletes may use vitamin D supplementation to cover their needs. Athletes with insufficient status require supplementation with 2000–4000 IU/day vitamin D to keep 25(OH)D concentrations in the sufficient range [52].

Although iron and vitamin D deficiencies are well documented among endurance athletes, there is no longitudinal data available for elite biathletes. Therefore, supplementation practices are largely empirical rather than evidence-based.

## 4. Physique and Performance in Biathlon

The world’s leading biathletes demonstrate high maximal oxygen uptake (VO_2_max), with males exhibiting values above 80 mL/kg/min and females above 65 mL/kg/min [53]. The most successful competitors are well-trained endurance athletes who demonstrate excellence in skating technique and can also compete at a high level in elite cross-country skiing events [1].

In existing research, the authors have demonstrated a correlation between body composition and physical performance among adolescents engaged in cross-country skiing [54,55]. It has been observed that athletes with lower body fat have higher maximal aerobic capacity (VO_2_max) [55], and thus should achieve superior results [56]. However, the results of different research studies indicated that high body mass may be beneficial for performance in cross-country skiing, particularly on the downhill section of the track. This study harmed performance due to body fat mass [54]. Notably, the ski course in this study was 5.6 km, which is shorter than any distance in a biathlon race. This may also explain why high lean body mass, particularly in the arms, was positively correlated with the overall time [54]. In a single study, the authors investigated the body mass of Norwegian biathletes who participated in the Olympic Games and World Championships between 1990 and 2013. The analysis revealed no statistically significant difference in body mass between biathletes who achieved a podium position and those who did not for both male and female competitors [53].

The biathlon can be divided into three parts, which can be considered as an overall result: skiing time (skiing speed), shooting accuracy, and range time. While body mass and body composition may impact performance during the ski running course, they should be neutral for shooting performance. Based on data from 17 seasons (2002/2003–2018/2019), the authors hypothesise that shooting is the most influential factor for biathlon performance [57]. Considering the aforementioned factors, it can be posited that there is no singular, optimal body mass and body composition for biathlon performance, provided that it aligns with the general recommendations for endurance sports [58].

Although body composition correlates with endurance performance, evidence from biathlon suggests that shooting proficiency is equally, if not more, important. Therefore, focusing solely on optimising physique could mean overlooking important factors for success.

## 5. Nutrition for Racing

Biathlon races are characterised by high-intensity effort lasting for 20–50 min. A Biathlon competition consists of several racing days. Based on the physiological demands, there is no need to use a carbohydrate-loading strategy to maximise glycogen stores. It has been proved that glycogen is not the limit factor for biathlon events with a duration of fewer than 90 min. However, biathlon events entail daily races when the recovery period is limited to less than 24 h. In such circumstances, the optimisation of carbohydrate intake during the post-exercise recovery period is of paramount importance. Table 3 illustrates nutrition periodization over the competition week.

Biathletes should focus on a carbohydrate-rich diet in the days before the competition and on race day. A low-fat, high-fibre, high-carbohydrate breakfast is recommended on race day, followed by a high-carbohydrate snack in liquid or solid form, depending on the time of ingestion relative to the race time. Despite the risk of reactive hypoglycaemia if carbohydrates are consumed in the last hour before the competition, performance has no detrimental effect [59]. More importantly, research on XC skiers suggests that a carbohydrate snack before a 15 km/20 km race for women or men can improve performance [60]. If an athlete is concerned about symptoms of hypoglycaemia, late ingestion of carbohydrates (a few minutes before exercise or during warm-up) is recommended.

During biathlon competitions, there is no need to consume carbohydrates or fluids. However, there is a general rule of thumb for the longest races, which is for both men and women to provide bottles of fluids. The transfer from the arena to the hotel and the various duties of the athletes (e.g., doping control, obligatory visit to the press area) usually delay the opportunity for the first meal. Therefore, a recovery shake containing ∼0.3 g of protein and ∼1.2 g/kg BM of carbohydrates is recommended immediately after the race. A liquid meal rehydrates the athlete and is better tolerated, even if appetite suppression occurs. If there is a need for a solid meal during World Cup or championship events, biathletes can use a “Family Club”, where athletes and staff can choose between different types of food or liquids. However, the food quality varies, depends on the organising committee and does not always meet the athlete’s requirements. Biathletes usually, but not always, have a day off between races, which allows them to replenish their glycogen stores fully. Table 4 provides nutritional recommendations for competitions that consider the sport’s unique logistical challenges, such as limited access to food and the need to recover within 24 h of racing.

Although general endurance race strategies can be applied to biathlon, logistical barriers such as doping control, media duties and limited access to food can hinder recovery. This highlights the importance of conducting studies that replicate real competition contexts.

## 6. Cold and High-Altitude

The biathlon is an outdoor discipline, and the associated cold temperatures, in addition to which athletes often spend the preparation period at high altitude, present a challenging environment for the athletes. The initial challenge is the surge in energy demand when these environmental conditions are considered. This is because metabolic heat production cold temperatures is at least doubled to maintain the body’s core temperature [61]. Furthermore, there is a notable shift in energy substrate utilisation at elevated altitudes with a greater reliance on blood glucose at rest and during exercise [62,63]. It can, therefore, be surmised that an adequate intake of energy and carbohydrates will prove beneficial in maintaining the weight and muscle mass of athletes in cold and high-altitude conditions [64]. Another challenge of training at altitude is the increased respiratory water loss and diuresis that is caused by hypoxia and low humidity in altitude exposure [65]. This is associated with increased water demand and reduced thirst and fluid availability during training [66,67]. Therefore, athletes should pay particular attention to monitoring their hydration status in high-altitude conditions, for example, by monitoring urine colour and daily weight change. Moreover, adequate hydration during training and between sessions is ensured [65].

Furthermore, it has been demonstrated that altitude exposure elevates erythroid iron requirements by a factor of three to five [68], exacerbating the risk of iron deficiency and rendering the replenishment of exercise-related iron losses challenging. Therefore, screening blood tests should be conducted 3–6 weeks before altitude exposure. These tests should comprise a complete blood count (in particular, a measurement of haemoglobin concentration), an iron profile (including serum iron, serum ferritin and transferrin saturation) and an assessment of C-reactive protein [46]. If the results indicate a ferritin level of less than 35 μg/L, a haemoglobin concentration exceeding 115 g/L, and a transferrin saturation greater than 16%, this indicates iron deficiency [69]. It is therefore recommended that oral iron supplementation of 200 mg for 2–6 weeks before and during altitude exposure [70] be undertaken by athletes in order to ensure that they maintain an adequate iron balance, which suggests an important role in the adaptation process. It is important to note, however, that iron supplementation should be initiated by the sports physician following a comprehensive review of the athlete’s blood profile prior to exposure.

Although it is clear that altitude and cold exposure alter energy, hydration and iron requirements, the absence of biathlon-specific data means that general endurance research must be relied upon. However, this research may not capture the combined effect of these stressors and rifle shooting.

## 7. Supplements and Sports Foods

**Figure 1:**
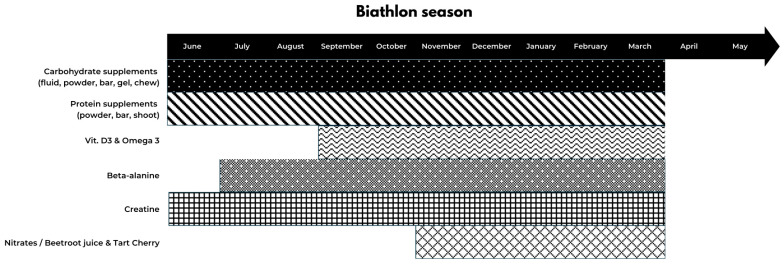
Supplements periodisation.

To date, we do not have specific data on biathletes’ use of dietary supplements. Complex information on dietary supplements can be found elsewhere [71]. The following section presents a series of recommendations regarding dietary supplements based on their mechanism of action and potential benefits for biathletes. Figure 1 represents supplement periodization throughout the year.

### 7.1. Caffeine

Caffeine is the most popular stimulant and has been shown to improve performance by reducing fatigue and enabling sustained power output. The effect of caffeine supplementation varies depending on the athlete’s genotype. Some individuals are non-responders, and others may react negatively to caffeine ingestion. Current recommendations suggest intakes of 3–6 mg/kg body mass [72], usually 30–60 min before competition. Studies conducted in cross-country skiing have shown reduced time to complete a set distance [73] and improved time to task failure [74]. However, caffeine supplementation negatively affected shooting performance during simulated biathlon competitions and tended to improve skiing performance [75]. This highlights that direct transfer of findings from other endurance sports can be misleading if the unique precision demands of biathlon are ignored.

### 7.2. Beta-Alanine

Long-term (4 to 24 weeks) supplementation with ß-alanine (4–6 g/d) increases muscle carnosine content [76], which improves buffering capacity [77]. Beta-alanine supplementation improves high-intensity endurance performance across a range of exercise capacity tests, fixed duration and intermittent exercise tasks, typically 30 s to 10 min in duration [77]. Although biathlon events last longer than 10 min, beta-alanine supplementation improves performance when high-intensity effort(s) are performed within or at the end of prolonged exercise. Long-term supplementation (up to 24 weeks) is safe; paraesthesia is the only side effect.

### 7.3. Creatine

Creatine is the most studied ergogenic supplement with a wide range of uses. One benefit is improving sprinting during or after endurance exercise, typical of biathlon competitions. Creatine supplementation increases power output and speed, reducing fatigue and the time needed to complete a given distance [78]. However, one of the side effects is rapid weight gain, which can be detrimental to biathletes’ performance, especially during ascents. It is recommended to take 3–5 g of monohydrated creatine daily.

### 7.4. Bicarbonate

Prior to the Single Mixed Relay, the shortest biathlon event, athletes can use bicarbonate supplementation. Bicarbonate is an endogenously produced extracellular anion that works by removing excess hydrogen ions produced during high-intensity exercise and, to some extent, supports the body’s ability to meet the high rates of energy demand required to maintain muscle contractile function during such activity. Oral ingestion of 200 and 300 mg/kg BM of sodium bicarbonate is sufficient to increase endogenous bicarbonate levels, which can improve performance by 2–3% during single or repeated bouts of high-intensity exercise, typically lasting 1–10 min [79,80,81]. However, it should be noted that there is a high risk of gastrointestinal upset with bicarbonate supplementation.

### 7.5. Dietary Nitrates

Nitrate supplementation has a wide range of effects on health and performance. From an athlete’s perspective, it may regulate blood pressure and blood flow, mitochondrial respiration, muscle contraction and immune function. Nitrate supplementation can improve running efficiency by reducing the absolute O_2_ cost for a given work rate [82] and improve endurance performance [83]. The full ergogenic effect and mechanism of action of nitrate supplementation are described elsewhere [84,85]. Taking a dose of 6–8 mmol (~350–500 mg) of nitrate (1–2 commercially available tablets) 2–3 h before the race, with or without supplementation for 3–7 days before the competition. However, it should be added that in well-trained athletes with VO_2_peak values >64.9 mL/kg/min, the improvement is not observed. It is worth reconsidering the supplementation strategy during training or less important races because of possible gastrointestinal side effects. It is advisable to avoid using mouthwash or chewing gum containing beetroot juice, as they may interfere with its benefits.

### 7.6. Tart Cherry

During the season, biathletes take part in nine World Cup competitions. Each of these takes place in a different location, so they sleep in a different hotel each time. Based on the preliminary research, some evidence supports the use of tart cherry to improve sleep duration and efficiency, presumably as an important source of melatonin [86]. In addition, tart cherry may be beneficial when repeated performance is required within a day or over several days, such as in biathlon [87]. Taking 45–100 cherry equivalents (e.g., 30 mL tart cherry juice) twice a day for 4–7 days before and during the competition period is recommended.

An important consideration in biathlon is that nutritional and supplementation strategies may not consistently affect the two key performance factors: skiing speed and shooting accuracy. As we mentioned above, caffeine is known to improve endurance by reducing perceived exertion and sustaining power output. However, the only study conducted in biathlon revealed impaired shooting accuracy despite improved skiing times. This illustrates a unique challenge in biathlon: a supplement that improves aerobic capacity may also compromise fine motor precision. Other ergogenic aids, such as nitrates, creatine and β-alanine, have primarily been studied in the context of endurance or intermittent high-intensity activities, and they may offer potential benefits for skiing. However, their influence on shooting performance remains unknown. Conversely, supplements that promote neuromuscular stability, reduce tremor or improve sleep quality (e.g., tart cherry juice, which contains melatonin) could indirectly support shooting performance, but the evidence is preliminary. Therefore, a cautious and individualised approach is required in biathlon, where both endurance and precision outcomes must be evaluated before practical recommendations can be made.

Moreover, it is important to remember that the use of dietary supplements is associated with the risk of a positive doping test. To minimise this risk, choosing supplements with a specific certificate, such as the Informed-Sport certificate or supplements included in the Cologne List, is advisable.

## 8. Summary

The nutritional requirements for biathletes differ from those for athletes in other endurance sports because they must sustain high levels of aerobic power while skiing and also maintain fine motor control and psychological composure while shooting. The training plan comprised various training sessions, characterised by various durations and intensities, which afforded particular energy and carbohydrate requirements. Athletes need to develop the ability to adapt their nutritional intake to the specific demands of each training session, the training week as a whole, and the overall period leading up to the event. The provision of carbohydrates in snacks before, during and after training sessions allows athletes to meet their energy and carbohydrate requirements. It is recommended that biathletes prioritise the development of lean body mass while maintaining a low but healthy level of adipose tissue. Nutrition during race day presents a logistical challenge; therefore, athletes should plan their meals and ensure the availability of carbohydrate snacks and recovery drinks in their bags. The recovery process should be prioritised immediately following the race. Since some supplements (e.g., caffeine) may enhance skiing but impair shooting accuracy, supplementation strategies require careful individual testing.

At the same time, this review highlights significant research gaps. No direct studies have been conducted on energy expenditure, macronutrient periodisation, recovery between consecutive races or the effects of caffeine supplementation in biathlon. Most recommendations are extrapolated from cross-country skiing, cycling or triathlon—disciplines which do not capture the combined physiological and technical demands of the biathlon. Future research must adopt a dual-performance framework that tests how nutrition influences endurance and precision outcomes under realistic training and competition conditions.

In summary, while evidence from related sports can offer valuable insights into current practices, the field of biathlon nutrition remains under-researched. Until sport-specific data becomes available, practitioners must carefully adapt general endurance nutrition principles to the sport’s unique physiological, technical, and logistical demands.

## Figures and Tables

**Table 1:** An example of a training week is provided for the summer period.

	Monday	Tuesday	Wednesday	Thursday	Friday	Saturday	Sunday
AM session	Complex (Z2)–140′	Complex (Z2/Z3)–140′	Cross–60′	Complex (Z2)–160′	Gym–120′	Roller skies (Z2/Z4)–120′	Complex (Z2/Z3)–120′
Session nutrition focus	CHO intake to maintain a stable blood glucose	HCHO-A and CHO intake to maintain a stable blood glucose		CHO intake to maintain a stable blood glucose	Protein	LCHO-A	HCHO-A and CHO intake to maintain a stable blood glucose
PM session	Gym–120′	Cross + core –100′	OFF	Cross + core –100′	Complex (Z2) –130′	Cross + core –80′	Cross + core –80′
Session nutrition focus	Protein				CHO intake to maintain a stable blood glucose		
Examples of daily nutrition strategies	CHO and protein recovery meal for glycogen resynthesis and muscle repair after the sessions and throughout the day	CHO intake during AM session for central drive and avoid impairment of neuromuscular precision	Reduced energy and CHO to meet low requirements	CHO intake during AM session	Protein snack post Gym session and restricted CHO refuelling after PM session for LCHO-A for AM session the next day	CAF supplementation before AM session; CHO recovery after AM session and throughout the day	CHO recovery and throughout the day
Biathlon-specific notes	Shooting in AM session included → avoid hypoglycemia to preserve fine-motor accuracy	Shooting in AM session included → avoid hypoglycemia to preserve fine-motor accuracy		Shooting in AM session included → avoid hypoglycemia to preserve fine-motor accuracy	Shooting in PM session included → avoid hypoglycemia to preserve fine-motor accuracy	No rifle; opportunity for metabolic adaptation	Shooting in AM session included → avoid hypoglycemia to preserve fine-motor accuracy

Notes: CAF–caffeine; CHO–carbohydrates; Complex–training consisting of roller skiing/cross-country skiing + shooting (precision + endurance required); Cross–running on different terrain; HCHO-A–high carbohydrate availability; LCHO-A–low carbohydrate availability; Z2–zone 2; Z3–zone 3; Z4–zone 4.

**Table 2:** An example of a training week is provided for the winter period.

	Monday	Tuesday	Wednesday	Thursday	Friday	Saturday	Sunday
AM session	Complex (Z2) –120′	Complex (Z2/Z3)–120′	Skies (Z2) 120′	Skies (Z2/Z3)–120′	Skies (Z2) –90′	Complex (Z2/Z3) –120′	OFF
Session nutrition focus	HCHO-A or CHO central	HCHO-A and CHO intake to maintain a stable blood glucose		LCHO-A Protein		HCHO-A and CHO intake to maintain a stable blood glucose	
PM session	Skies (Z2)–90′	Cross + core –60′	OFF	Complex (Z2) –100′	Complex (Z2) –90′	Cross + core –60′	OFF
Session nutrition focus				CHO intake to maintain a stable blood glucose	CHO intake to maintain a stable blood glucose		
Examples of daily nutrition strategies	CHO intake during AM session for central drive and avoid impairment of neuromuscular precision Session; CHO recovery after AM session due to second long session	CHO intake during AM session for central drive and avoid impairment of neuromuscular precision	Restricted refuelling after AM session for LCHO-A for AM session the next day	CHO Recovery after sessions and throughout the day due to second long session	CHO Recovery after sessions and throughout the day due to second long session	CHO intake during AM session	Reduced energy and CHO to meet non-training requirements
Biathlon-specific notes	Shooting in AM session included → avoid hypoglycemia to preserve fine-motor accuracy	Shooting in AM session included → avoid hypoglycemia to preserve fine-motor accuracy		No rifle in AM session → opportunity for metabolic adaptation; Shooting in PM session included → avoid hypoglycemia to preserve fine-motor accuracy	Shooting in PM session included → avoid hypoglycemia to preserve fine-motor accuracy	Shooting in AM session included → avoid hypoglycemia to preserve fine-motor accuracy	

Notes: CAF–caffeine; CHO–carbohydrates; Complex–training consisting of roller skiing/cross-country skiing + shooting (precision + endurance required); Cross–running on different terrain; HCHO-A–high carbohydrate availability; LCHO-A–low carbohydrate availability; Z2–zone 2; Z3–zone 3; Z4–zone 4.

**Table 3:** An example of a training week is during the winter period.

	Monday	Tuesday	Wednesday	Thursday	Friday	Saturday	Sunday
AM session		Complex (Z1) –80′	Complex (Z1) –40′	Official training (Z1)–70′	Cross–40′	Cross–40′	Cross–40′
Session nutrition focus							
PM session	Skies (Z1 + sprints) the–60′	Cross + core –50′		Cross + core –50′	Race (Sprint) 10 km	Race (Pursuit) 12.5 km	Race (Relay) 4 × 7.5 km
Session nutrition focus					HCHO-A	HCHO-A	HCHO-A
Examples of daily nutrition strategies	Reduced energy and CHO to meet low requirements		Reduced energy and CHO to meet low requirements	High CHO snack; normal level of CHO during the day to normalise muscle glycogen level	CHO snack before the race; CHO throughout the day and recovery	CHO snack before race; CHO throughout the day and recovery	CHO snack before race; CHO throughout the day and recovery
Biathlon-specific notes		Shooting in AM session included → avoid hypoglycemia to preserve fine-motor accuracy	Shooting in AM session included → avoid hypoglycemia to preserve fine-motor accuracy	Shooting in AM session included → avoid hypoglycemia to preserve fine-motor accuracy	CHO snack a few minutes before the start; recovery shake immediately after the finish to begin the recovery process	CHO snack a few minutes before the start; recovery shake immediately after the finish to begin the recovery process	CHO snack a few minutes before the start

**Table 4:** Nutrition recommendation for competition.

The Race Starts Before 14.00				
	Time	Recommendation	Strategies	Example
Breakfast	8.00–9.00	Reduce the weight of gut contents to reduce BM; reduce the risk of gut discomfort during racing; optimise liver glycogen stores.	Replace wholemeal and unrefined grains with ‘white’ or ‘refined’ options (e.g., white bread, white rice, pasta). Eliminate raw and skinned vegetables from meals and recipes. Limit vegetables to small amounts that can be eaten peeled, well-cooked and mashed. Consume 1–4 g/kg carbohydrates 1–4 h before start. If you have a positive GI history, replace HIGH FODMAP products (e.g., lactose, apple, cherry, wheat, dates, protein powders, sugar-free products) with LOW FODMAP options (e.g., lactose-free milk, banana, orange, gluten-free products, rice, non-polyol products).	Large bowl of cereal with skim milk, lactose-free milk, banana, glass of orange juice. Pancakes with melon, low-fat, lactose-free yoghurt, and glass of orange juice. Rice pudding with berries and almonds, glass of orange juice. Baguette with low-fat mozzarella, tomato, glass of orange juice.
Snack	11.00–12.00	Optimise liver glycogen stores to avoid hunger.	Consume 30–60 g of carbohydrates 5–90 min before the start or during warm-up.	Banana Sports energy bar/gel/chew/drink Bun with jam
Post race	14.00–15.00	Replenishment of glycogen stores and hydration status.	Consume 1.0–1.5 g/kg carbohydrates with 20–30 g of protein, avoid high amounts of fat and fibre.	CHO-PRO recovery shake, energy bar. Milkshake with banana and protein powder, sports drink. Sandwich with ham and low-fat cheese, a glass of juice.
Lunch	15.30–16.30	Replenishment of glycogen stores.	Consume 1.0–1.5 g/kg of carbohydrates and 20–40 g of protein. Choose products rich in antioxidants.	Pasta with tomato sauce and low-fat cheese, fruit. Baked potato with grilled vegetables and chicken breast, glass of juice. Rice with vegetable stew, glass of juice.
Dinner	19.00–20.00	Continue to eat as normal until reaching daily macronutrient targets.	Consume 1.0–1.5 g/kg carbohydrates with 20–40 g of protein.	Spaghetti Bolognese, a vegetable salad, a glass of juice. Risotto with lentils, mixed salad with vinaigrette souse, and a fruit bowl. Grilled salmon with rice, fresh vegetable salad, and a glass of juice.
The race starts after 14.00				
Breakfast	9.00	Reduce the weight of gut contents to reduce BM; reduce the risk of gut discomfort during racing; optimise liver glycogen stores.	Replace wholemeal and unrefined grains with ‘white’ or ‘refined’ options (e.g., white bread, white rice, pasta). Eliminate raw and skinned vegetables from meals and recipes. Limit vegetables to small amounts that can be eaten peeled, well cooked and mashed. Consume 1–4 g/kg carbohydrates 1–4 h before start. If you have a positive GI history, replace HIGH FODMAP products (e.g., lactose, apple, cherry, wheat, dates, protein powders, sugar-free products) with LOW FODMAP options (e.g., lactose-free milk, banana, orange, gluten-free products, rice, non-polyol products).	Large bowl of cereal with skim milk, lactose-free milk, banana, glass of orange juice. Pancakes with melon, low-fat, lactose-free yoghurt, and glass of orange juice. Rice pudding with berries and almonds, glass of orange juice. Baguette with low-fat mozzarella, tomato, and a glass of orange juice.
Lunch	12.00–13.00	Reduce the weight of gut content to reduce BM; reduce the risk of gut discomfort during the race.	Replace wholemeal and unrefined grains with ‘white’ or ‘refined’ options (e.g., white bread, white rice, pasta). Eliminate raw and skinned vegetables from meals and recipes. Limit vegetables to small amounts that can be eaten peeled, well-cooked and mashed. Consume 1–4 g/kg carbohydrates 1–4 h before start. If you have a positive GI history, replace HIGH FODMAP products (e.g., lactose, apple, cherry, wheat, dates, protein powders, sugar-free products) with LOW FODMAP options (e.g., lactose-free milk, banana, orange, gluten-free products, rice, non-polyol products).	Pasta with tomato sauce. Rice with parmesan and zucchini. Rice pudding with berries and almonds, a glass of orange juice.
Snack	15.00–16.00	Optimise liver glycogen stores to avoid hunger.	Consume 30–60 g of carbohydrates 5–90 min before the start or during warm-up.	Banana Sports energy bar/gel/chew/drink Bun with jam
Post-race	17.00–18.00	Replenishment of glycogen stores and hydration status.	Consume 1.0–1.5 g/kg carbohydrates with 20–30 g of protein and avoid high amounts of fat and fibre.	CHO-PRO recovery shake, energy bar. Milkshake with banana and protein powder, sports drink. Sandwich with ham and low-fat cheese, a glass of juice.
Dinner	19.30–20.30	Continue to eat as normal until reaching daily macronutrient targets.	Consume 1.0–1.5 g/kg carbohydrates with 20–40 g of protein.	Spaghetti Bolognese, a vegetable salad, and a glass of juice. Risotto with lentils, a salad mix with vinaigrette souse, and a bowl of fruits. Grilled salmon with rice, fresh vegetables salad, and a glass of juice.

## Data Availability

Not applicable.
